# Case Report: The first account of primary myeloid sarcoma of the testis combined with ipsilateral epididymal epithelioid hemangioendothelioma

**DOI:** 10.3389/fmed.2025.1590907

**Published:** 2025-09-05

**Authors:** Xi Chen, Li Liu

**Affiliations:** Department of Pathology, Second Affiliated Hospital of Chongqing Medical University, Chongqing, China

**Keywords:** myeloid sarcoma, testis tumors, epithelioid hemangioendothelioma, epididymis tumors, immunohistochemistry, WWTR1-CAMTA1 gene

## Abstract

This case report describes a 35-year-old male with primary myeloid sarcoma (MS) of the testis and concurrent ipsilateral epididymal epithelioid hemangioendothelioma (EHE)—an exceptionally rare combination. Myeloid sarcoma typically involves the skin and bone, but testicular involvement is rare, particularly as an isolated tumor without hematologic malignancy. This makes diagnosis challenging. Epithelioid hemangioendothelioma is a rare vascular tumor, typically found in the liver, lungs, and bones, and epididymal EHE is scarcely reported. The patient had a left testicular nodule for 6 months and experienced pain for 2 days. Ultrasound and MRI revealed abnormalities in the left testis and epididymis. Histopathological examination of the epididymal biopsy showed tumor cells with specific morphological features, and immunohistochemistry (IHC) indicated CD31(+), CD34(+), Fli-1(+), ERG(+), Ki-67(+5%+), along with WWTR1-CAMTA1 gene fusion by fluorescence *in situ* hybridization (FISH), confirming EHE. Subsequently, testicular resection was performed, and the testicular tumor cells were diffusely arranged. Immunohistochemistry (IHC) analysis revealed expression of multiple markers such as CD31, CD34, MPO, LCA, CD99, CD117, and Ki-67(+50%+), leading to the diagnosis of MS. The patient was then treated with the MA chemotherapy regimen. Diagnosing MS requires integration of clinical history, histopathology, and IHC, but misdiagnosis is common due to overlapping features with other malignancies. There is no standard treatment for testicular MS, but early diagnosis is critical. EHE is mainly treated by extensive resection. This case highlights the importance of suspecting MS in differential diagnosis of testicular and epididymal tumors and calls for further research on the potential interaction between MS and EHE in tumorigenesis.

## 1 Introduction

A tumor mass consisting of myeloid blasts, occurring at a site other than the bone marrow, is diagnosed as myeloid sarcoma (MS) (1). In 1811, MS was described for the first time by Burns. Afterward, in 1853, King called the disease chloroma due to its greenish appearance (2). It has been reported in 2–8% of patients with acute myeloid leukemia (AML) and is commonly diagnosed simultaneously with AML (3). MS may also be associated with myeloproliferative neoplasm or myelodysplastic syndrome (4–6). In addition, it can present as an isolated tumor with no evidence of hematological malignancy (isolated MS). The most common sites of involvement of myeloid sarcoma include the skin, bone, lymph node and soft tissues (7). The testicles are considered a rare site (8–10). Because of the absence of blood and marrow manifestations, isolated MS is a diagnostic challenge and is often misdiagnosed as another disease, such as a lymphoma or vascular tumor (11).

Epithelioid hemangioendothelioma (EHE) is a rare vascular tumor with an incidence of one in a million, according to the National Institutes of Health (NIH) in the United States. Only 20 cases are diagnosed annually in the US, with the average age of ranging from 30 to 50 years, and a predominance in females (12). The Hemangioendothelioma and Related Vascular Disorders (HEARD) international registry has gathered the most comprehensive data on EHE and its progression: among 206 patients, the most common sites of EHE involvement are the liver (21%), liver and lung (18%), lung (12%), and bone (14%) (13). However, epididymal EHE is extremely rare, and there are no reports of it occurring concurrently MS of the ipsilateral testicle. MS and EHE demonstrate significant differences in biological characteristics, treatment strategies, and prognosis. As a rare hematological malignancy, MS typically requires initial treatment with AML chemotherapy regimens containing cytarabine, while radiotherapy and surgery serve as adjuvant modalities. In contrast, EHE is a low- to intermediate-grade malignant vascular tumor with potential metastatic capacity, primarily managed by complete surgical resection. The therapeutic approaches for these two tumors are fundamentally distinct. Concurrent occurrence of the two tumors highly predisposes to misdiagnosis or missed diagnosis. Clinically, the rapidly progressive mass and systemic symptoms of MS may interfere with the insidious growth and indolent course of EHE. Pathologically, their histomorphological features may be confounded by sampling bias or atypical manifestations. Additionally, cross-expression of immunohistochemical markers (e.g., partial overlap between myeloid markers in MS and vascular endothelial markers in EHE) further complicates accurate diagnosis.

Delayed identification entails severe clinical consequences: Missed diagnosis of MS may forfeit the optimal window for intensive chemotherapy and transplantation, accelerating progression to fatal hematological malignancies. Neglect of EHE can lead to local tumor residue and recurrence, compromising long-term quality of life. Inappropriate treatment selection not only delays disease management but also risks severe adverse reactions from mismatched therapies, endangering patient safety. Therefore, clinicians must remain highly alert to such rare co-occurrences, leveraging multidisciplinary collaboration, comprehensive pathological assessment, and molecular testing to minimize diagnostic errors and formulate precision treatment strategies.

We herein report a case of a primary MS of the testicle combined with ipsilateral epididymal EHE along with relatively detailed clinicopathological information to provide more data for rare diseases.

## 2 Case description

**Figure 1:**
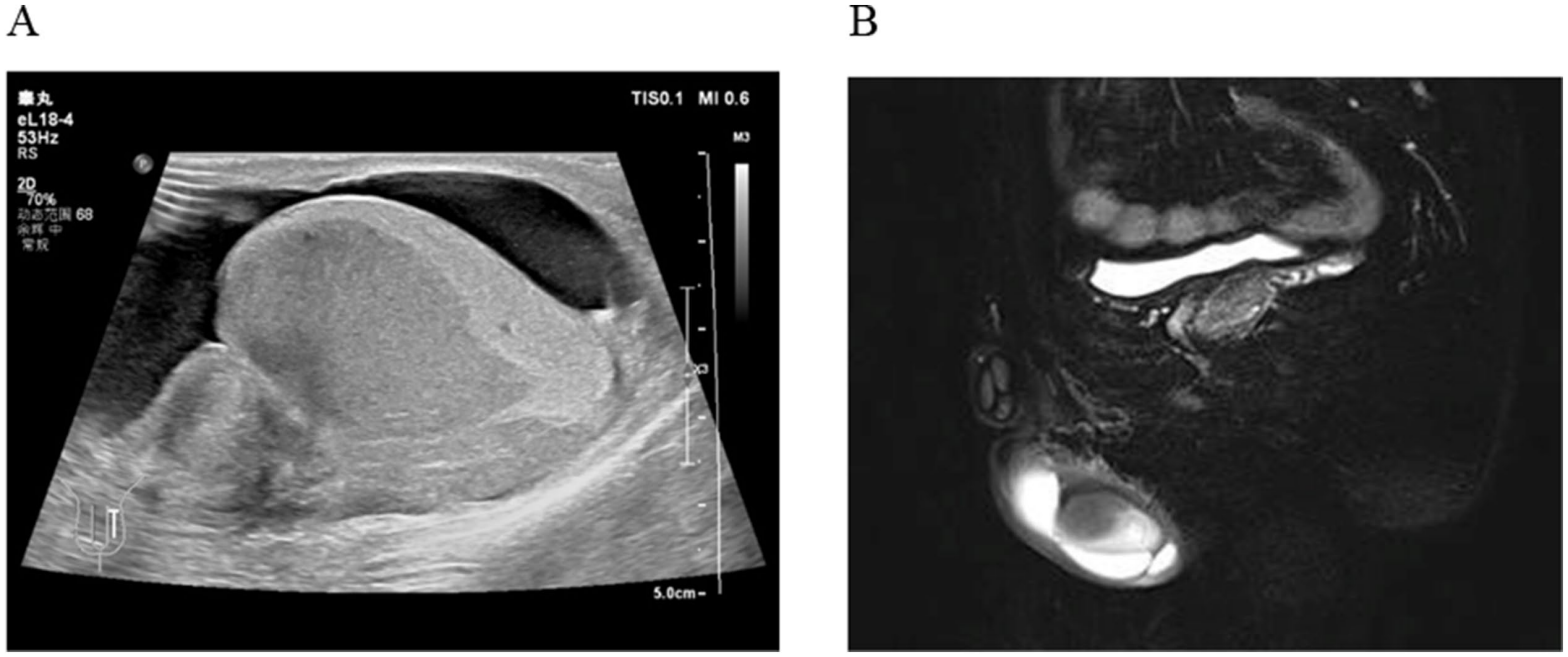
**(A)** Ultrasound shows a hypoechoic area in the left testicle; Enlarged left epididymis with abundant blood supply. **(B)** MR shows patchy abnormal signals in the left epididymis and testes.

A 35-year-old man was admitted to Department of Urology on April 27, 2024, due to the discovery of a nodule behind the left testicle for 6 months, accompanied by pain for 2 days. Ultrasound shows a hypoechoic area in the left testicle, further examination is recommended. Enlarged left epididymis with abundant blood supply. There is a large amount of fluid accumulation in the left testicular sheath cavity (Figure 1A). Magnetic resonance imaging (MR) shows patchy abnormal signals in the left epididymis and testes, suggesting the possibility of infectious lesions; Bilateral testicular hydrocele (Figure 1B). He had a 10-year history of smoking (approximately 20 cigarettes per day) and no known family history of cancer, genetic diseases, or a history of long-term medication use. Additionally, there was no evidence of current or previous soft tissue tumors or history of radiation exposure.

**Figure 2:**
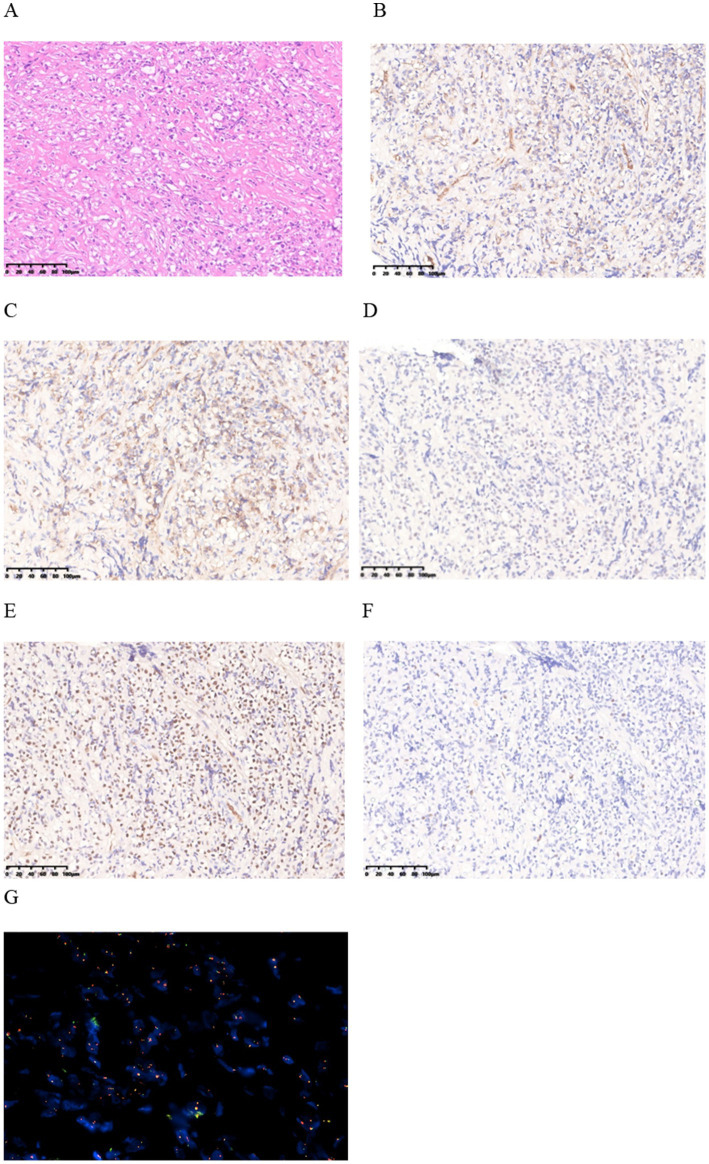
**(A)** HE staining finding at 20x magnification. **(B–E)** Immunohistochemical staining shows positive staining for CD31, CD34, Fli-1, and ERG in the lesion. **(F)** Immunohistochemical staining for Ki-67 shows a low proliferation index with 5% positivity. **(G)** FISH revealed fusion of WWTR1-CAMTA1 gene.

Subsequently, an incision and exploration of the scrotum and biopsy of the left epididymis were performed. During the operation, it was observed that the head of the epididymis was significantly enlarged, with pale tissue, a tough texture, and an unclear boundary with the testis. The histomorphological manifestation is composed of tumor cells arranged in patches, which are round in shape and have vacuoles formed in the cytoplasm. The cellular atypia is mild, and no clear signs of nuclear division are observed (Figure 2A).

Immunohistochemical (IHC) analysis of the epididymal lesions further revealed cytokeratin (CK)(−), vimentin (−), CD31(+), CD34(+), Fli-1(+), ERG(+), Ki-67(+5%+) (Figures 2B–F). Fluorescence *in situ* hybridization (FISH) revealed fusion of WWTR1-CAMTA1 gene (Figure 2G). The diagnosis of EHE was eventually made by consultation with immunohistochemical and FISH. This comprehensive analysis played a crucial role in elucidating the underlying pathology and guiding subsequent steps in patient management.

**Figure 3:**
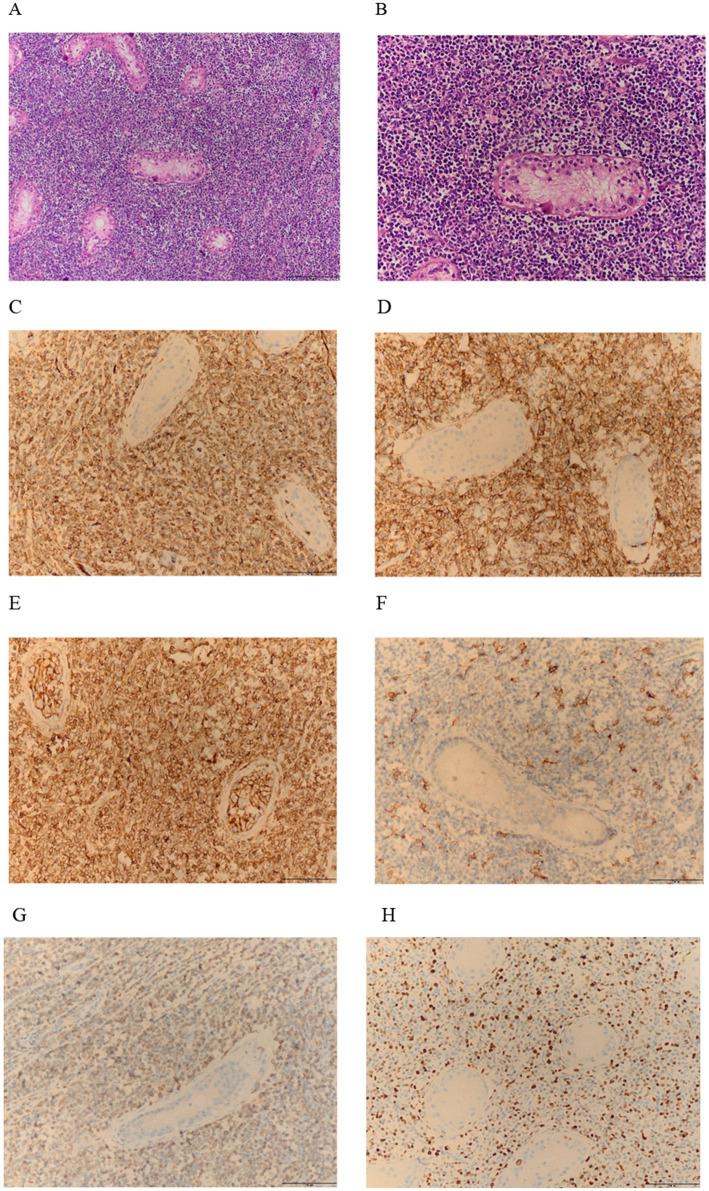
**(A,B)** HE staining shows numerous atypical cells: at 10x and 20x magnification. **(C–G)** Immunohistochemistry indicates that CD31, CD43, CD99, and MPO are positively expressed within the lesion **(C–E,G)**; CD163 is positive in small areas **(F)**; **(H)** The Ki-67 proliferation index is high, with a positivity rate of 50%.

**Figure 4:**
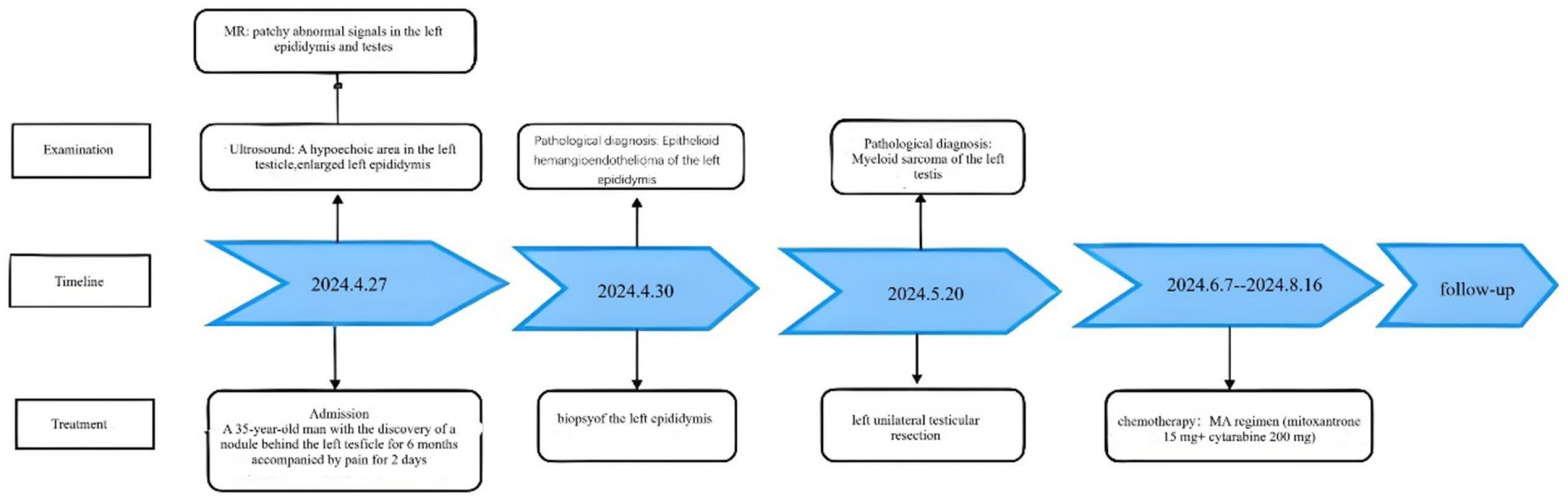
Timeline scheme of the major clinical event of the patient.

Two weeks later, the patient was readmitted for left unilateral testicular resection after excluding contraindications. Under the light microscope, tumor cells in the testes are diffusely arranged. The tumor cells are medium-sized or slightly large, round or oval in shape, with less cytoplasm and delicate nuclear chromatin. Small nucleoli can be seen, and nuclear division is easy to observe (Figures 3A,B). IHC analysis of the testicular tumor further revealed CK(−), vimentin(−), CD31(+), CD34(+), CD43(+), MPO(+), LCA(+), CD99(+), CD117(+), CD163(foci +), P53(>80%+), CD3(−), CD5(−), CD20(−), CD19(−), PAX5(−), Ki-67(+50%+) (Figures 3C–H), FISH did not detect the fusion of WWTR1-CAMTA1 gene. Pathological biopsy diagnosis is myeloid sarcoma. Pathological examination of blood cells showed no significant abnormalities, flow cytometry showed that primitive granulocytes accounted for 0.31%, and bone marrow cell examination showed active bone marrow proliferation. Therefore, the final diagnosis is primary MS of the testicle combined with ipsilateral epididymal EHE. The patient received one cycle of MA chemotherapy (mitoxantrone 15 mg + cytarabine 200 mg), followed by two high-dose cytarabine cycles (3 g every 12 h). At the same time, targeted supportive treatments were provided. Later, the patient’s condition stabilized, and he was discharged from the hospital and underwent regular follow–up (Figure 4). Since discharge, the patient has undergone regular follow-up for 9 months. Periodic flow cytometry, bone marrow cell examination, and pathological morphology analysis of blood cells have revealed no evidence of progression to AML. Chemotherapy-induced myelosuppression was treated with granulocyte colony-stimulating factor for leukocytosis and thrombopoietin for thrombocytopenia, and the condition has remained stable.

## 3 Immunohistochemistry and fluorescence *in situ* hybridization

IHC was performed in the Department of Pathology of the Second Affiliated Hospital of Chongqing Medical University. Initially, the slides undergo dewaxing and hydration. Subsequently, they are incubated in a 3% hydrogen peroxide solution for 10 min to block endogenous peroxidase activity. This step is followed by washing the slides three times with phosphate-buffered saline (PBS) at pH 7, each time for 3 min. The slides are then placed in a citrate buffer solution (pH 6.0) and subjected to microwave heating for antigen retrieval, twice, each time for 10 min. After heating, the slides are allowed to cool at room temperature. Subsequently, they are again washed three times with PBS at pH 7.4, each for 3 min. Depending on the tissue size, an appropriate amount of primary antibody is applied and incubated overnight at 4°C. After incubation, the slides are washed three times with PBS, each for 3 min. This is followed by the addition of an appropriate amount of enzymelabeled polymer goat anti-mouse/rabbit IgG; thereafter, the slides undergo incubation at room temperature for 20 min. After incubation, the slides are washed three times with PBS, each for 3 min. Finally, the slides are visualized using a freshly prepared diaminobenzidine chromogen solution. Post-visualization, the slides are rinsed with PBS and counterstained with hematoxylin. We added positive and negative controls during the experiment to demonstrate the validity and reliability of the staining process. IHC results are evaluated and interpreted by independent pathologists.

Next, the WWTR1-CAMTA1 fusion probe (Guangzhou LBP Medical Technology Co., Ltd., China) was used for interphase FISH, including rhodamine-conjugated in the distal region of WWTR1 that emits red fluorescence and fluorescein isothiocyanate conjugated at the proximal region of the CAMTA1 gene that emits green fluorescence. The slices (4 μM) were dewaxed with xylene, incubated at 56°C for 16 h and dehydrated with ethanol. All tissue slices were boiled in the pretreated solution for 30 min and then digested with protease K for 10 min according to the manufacturer’s instructions. After a second dehydration step, the probe was applied to the slice, and the covered slide was sealed with a rubber adhesive, thermally denatured and left to hybridize at 37°C for 16 h. All tissue slices were then restained with DAPI in the installation medium and observed under a fluorescence microscope. Negative controls were used in each case. When at least 10 (20%) of the 50 counted tumor cells showed (yellow) fusion signals, the case was interpreted as positive for the fusion gene.

## 4 Discussion

Myeloid sarcoma (MS) is a tumor mass consisting of myeloid blasts, with or without maturation, occurring at an extra medullary site (14). Very rarely MS can be the only site of disease. MS of rare sites like testes, ovaries, pancreas, breast, and shoulder, have also been reported in the recent past (15–18). Among the rare sites, testicular MS is more complex to manage because of additional associated fertility issues.

Goyal et al. reviewed 10-year data (2004–2013) from the National Cancer Database (NCDB)and reported 746 registered patients with a new diagnosis of MS (19). Of these, only 43 MS cases (5.8%) originated from the reproductive system. According to them, the median age of patients with MS involving the reproductive system was almost a decade younger (42 years) than the total of patients with MS (59 years). This case is a young 35-year-old male with early onset of disease, no prior history of acute myeloid leukemia (AML), myelodysplastic syndrome, or myeloproliferative disorders.

Ultrasound is useful in defining mass size and excluding epididymoorchitis as a differential, but lacks specificity and sensitivity to diagnose the type of malignancy. Testicular biopsy is a necessary means to diagnose testicular MS. In the present case, histopathological findings comprised overwhelming monotonous hyperchromatic cells with scanty cytoplasm and sparse eosinophilic myelocytes, a helpful diagnostic clue but not always found in other cases. Hence, myeloid sarcoma was the final diagnosis based on the histopathological findings, clinical history and immunohistochemistry.

A combination of clinical history, histopathological findings and a panel of antibodies in immunohistochemistry is mandatory for a correct diagnosis of myeloid sarcoma (20, 21). However, it is still challenging to distinguish myeloid sarcoma from other malignancies such as malignant lymphoma and other poorly differentiated carcinoma, predominantly due to the similar histopathological features on H&E sections. Immunohistochemical studies with a panel of antibodies were performed in this case and disclosed expression of lysozyme, CD68, CD34, CD117 (c-Kit), vimentin and leucocyte common antigen, but not of B cell-specific (CD20) or T cell-specific (CD3) antigens, cytokeratin, PAS and epithelial membrane antigen. Seminoma may also be considered as it is the most commonly encountered tumor in the testis (22). However, the lack of PAS immunoreactivity and the absence of clear and vacuolated cytoplasm excluded the possibility of seminoma (23, 24).

Even so, misdiagnosis remains a major issue for these cancers, as noted by McIlwain et al., where six of eight cases of reviewed testicular MS were initially misdiagnosed (9). The most common misdiagnoses are lymphoma, infection, spermatocele, and plasmacytoma. Flow cytometry’s increasing availability has made it easier to distinguish between morphological variants and further emergence of the use of cytogenetics as a diagnostic tool for hematological malignancies can have important prognostic and treatment indications (25).

Currently, there is no consensus on the optimal treatment for testicular MS. Management is often multimodal utilizing orchidectomy as a diagnostic and therapeutic tool, followed by a chemotherapeutic regimen and, in some cases, radiation therapy (RT). From the International Lymphoma Radiation Oncology Group (ILROG) recommended RT, in the following scenarios: (i) for patients with isolated MS and inadequate response to chemotherapy, (ii) with isolated recurrence after allo-HSCT, and (iii) for palliation of symptomatic vital structure compression (26). In isolated MS, systemic chemotherapy has been shown to decrease progression to AML and increase overall survival. Due to the highly aggressive nature of MS, an early diagnosis of testicular MS is associated with better survival outcomes for patients (27). The mean time to develop AML from the diagnosis of isolated MS may be 5 months, and as such it is typically recommended that extramedullary lesions are treated with upfront chemotherapy following an AML regimen immediately post diagnosis (19).

The MA regimen is one of the commonly used chemotherapy protocols for AML and falls within the category of standard induction chemotherapy regimens. When compared with other AML induction remission regimens such as the DA regimen (daunorubicin + cytarabine) and the IA regimen (idarubicin + cytarabine), mitoxantrone belongs to anthraquinone drugs (with a structure similar to anthracyclines), and its cardiotoxicity is lower than that of daunorubicin and idarubicin. Taking all factors into consideration, the patient in this case chose the MA regimen as the treatment approach.

Vascular tumors of the epididymis are rare, and both benign hemangiomas and malignant angiosarcomas have been reported. The findings and immunohistochemical staining and fluorescence *in situ* hybridization in this case support the diagnosis of EHE. EHE is a vascular endothelial cell-derived tumor with epithelioid cell features, first named by Weiss and Enzinger in 1982, and was initially considered an intermediate tumor. Due to its high recurrence, metastasis, and mortality rates, it was classified as a low-grade malignant tumor in 2002 and 2013 editions of the World Health Organization (WHO). EHE most often occurs in soft tissues, and when it occurs solid organs, it is relatively common in the lung, liver, and bone, while it is only reported in individual cases in the epididymis. EHE is a well-circumscribed nodular mass, and the tumor cells are epithelioid or spindle-shaped with abundant cytoplasm and some cells have vacuoles in the cytoplasm, and some vacuoles contain red blood cells, which are arranged in sheets, nests, or cords and the stroma may have myxoid degeneration. Immunohistochemical staining, tumor cells express vascular endothelial markers CD34, CD31, ERG and FLI-1, 25 to 30% of cases express CK (AE1/AE3 or CAM5.2) and EMA, Approximately 90% of EHEs have WWTR1-CAMTA1 gene fusion, and about10% of EHEs have YAP1-TFE3 gene fusion (28). In this case, FISH detected the WWTR1-CAMTA1 gene fusion.

EHE needs to be differentiated from the following diseases: (1) infectious lesions: often accompanied by obvious inflammatory reactions, such as of inflammatory cells such as neutrophils, lymphocytes, and plasma cells; (2) metastatic poorly differentiated carcinoma: it needs to be differentiated in combination with history, organ-specific immunohistochemical markers, and genetic changes; (3) epithelioid hemangioma: EHE cells show intracytoplasmic vacuoles, usually without obvious vascular differentiation, and show mucoid stroma and characteristic genetic changes; epithelioid hemangioma has obvious vascular formation and interstitial of various inflammatory cells such as eosinophils; (4) epithelioid angiosarcoma: it often forms a complex tubular structure, and the have obvious malignant signs, and mitotic figures are easily seen, lacking CAMTA1, WWTR1, and TFE3 gene changes; (5) epithelioid sarcoma: it does not express vascular endothelial cell markers, and has specific INI-1 expression loss; and (6) perivascular epithelioid tumor (PEComa): some PEComas also have TFE3 fusion gene changes, and the tumor cells of PEComa occurring in the lung have transparent cytoplasm, which is epithelioid, but there is no cytoplasmic vacuole, nuclear deviation, and red blood cells in the vacuole Immunohistochemical expression of HMB45, PNL2, Melan-A, and MiTF, etc., which are melanocyte markers, helps to distinguish.

The treatment of EHE is mainly based on extensive resection, and systemic therapy such as radiotherapy and chemotherapy is only used for patients with progressive after the original observation period and (or) with tumor-related symptoms or at high risk of organ dysfunction (29). The overall prognosis of EHE is relatively good with a 5-year survival rate of about 81% and a mortality rate of 5 to 20%. EHE occurring in the pleura aggressive and has a poor prognosis (30).

In conclusion, primary myeloid sarcoma of the testicle combined with ipsilateral epididymal epithelioid hemangioendothelioma is a rare case. We present the case not only due to its rarity, but also to guide physicians on the importance of a high index of suspicion for myeloid sarcoma in the differential diagnosis of other diseases, such as vascular tumors, poorly differentiated carcinoma, melanoma, T cell lymphoma, Hodgkin’s disease and non-Hodgkin’s large cell lymphoma, to render correct diagnosis and proper treatment (31, 32).

In this case, two distinct tumors arose: one in the testis and another in the epididymis, both of which were malignant and had a short history (6 months of discovery). Although the size of the testicular tumor is large, the sequence of occurrence of the two is not clear. And the pathogenesis of tumor is complex. The coexistence of MS and EHE in the same anatomical region may involve shared pathogenetic mechanisms: (1) microenvironmental crosstalk, where MS-derived VEGF/PDGF promotes angiogenesis via VEGF/VEGFR2/Ang-Tie pathways utilized by EHE, alongside overlapping immune evasion from Treg/MDSC aggregation and EHE’s immunosuppressive network; (2) genetic overlap, including RUNX1/C-KIT mutations in MS and WWTR1-CAMTA1 fusion in EHE, potentially cross-activating through epigenetic (e.g., histone modification) or PI3K-AKT–mTOR pathways, with shared TP53/PTEN inactivation driving uncontrolled proliferation; and (3) functional tumor-tumor interactions, where MS-derived IL-6/TNF-*α* activates NF-κB for EHE invasion while EHE-secreted Ang-1/2 enhances MS vascularization via Tie2, alongside synergistic ECM remodeling from myeloid sarcoma MMPs degrading basement membrane for EHE migration and EHE-induced vascular damage promoting myeloid cell infiltration. These mechanisms highlight potential integrated therapeutic targets (33–36). The pathogenesis still requires in-depth investigation.

## Data Availability

The raw data supporting the conclusions of this article will be made available by the authors, without undue reservation.
